# An FPGA-Based Reconfigurable Accelerator for Real-Time Affine Transformation in Industrial Imaging Heterogeneous SoC

**DOI:** 10.3390/s26010316

**Published:** 2026-01-03

**Authors:** Yang Zhang, Dejun Chen, Huixiong Ruan, Hongyu Jia, Yong Liu, Ying Luo

**Affiliations:** School of Optoelectronic Science and Engineering, University of Electronic Science and Technology of China, Chengdu 611731, China; 202422050233@std.uestc.edu.cn (Y.Z.); chendejun@uestc.edu.cn (D.C.); 202211050809@std.uestc.edu.cn (H.R.); jiahongyu@uestc.edu.cn (H.J.); yongliu@uestc.edu.cn (Y.L.)

**Keywords:** affine transformation, field programmable gate array (FPGA), hardware accelerator, heterogeneous systems, image correction, memory prefetching

## Abstract

Real-time affine transformation, a core operation for image correction and registration of industrial cameras and scanners, faces challenges including the high computational cost of interpolation and inefficient data access. In this study, we propose a reconfigurable accelerator architecture based on a heterogeneous system-on-chip (SoC). The architecture decouples tasks into control and data paths: the ARM core in the processing system (PS) handles parameter matrix generation and scheduling, whereas the FPGA-based acceleration module in programmable logic (PL) implements the proposed PATRM algorithm. By integrating multiplication-free design and affine matrix properties, PATRM adopts Q15.16 fixed-point computation and AXI4 burst transmission for efficient block data prefetching and pipelined processing. Experimental results demonstrate 25 frames per second (FPS) for 2095×2448 resolution images, representing a 128.21 M pixel/s throughput, which is 5.3× faster than the Block AT baseline with a peak signal-to-noise ratio (PSNR) exceeding 26 dB. Featuring low resource consumption and dynamic reconfigurability, the accelerator meets the real-time requirements of industrial scanner correction and other high-performance image processing tasks.

## 1. Introduction

Affine image transformation, a fundamental geometric transformation in computer vision and image processing, is critical for establishing spatial correspondence between image pixels [1]. It encompasses core operations such as rotation, translation, scaling, and shearing, enabling flexible adjustment of image geometry while preserving the collinearity and parallelism of the pixels [2,3,4]. Affine transformation is widely employed in image registration, fusion, and correction, with applications in remote sensing, medical imaging, and autonomous driving [5,6]. In real-time video fusion, affine transformation facilitates the registration of multispectral images [7]. In panoramic stitching, the alignment of overlapping regions is supported [8]. These applications underscore their role as the cornerstones of modern image processing.

The image correction process involves three key steps. These steps include determining the corresponding point pairs, calculating the affine transformation matrix, and performing affine transformation-based image interpolation [9]. The final step is the core of affine transformation. This step is highly computationally intensive as it involves trigonometric functions, matrix multiplication and interpolation. In image registration tasks, it dominates up to 85% of the total central processing unit (CPU) execution time [10]. Thus, latency reduction is essential for real-time performance in intensive image-processing tasks. FPGAs offer several advantages, including low latency, low power consumption, and high system flexibility [11]. These features have motivated extensive research in this area [12,13,14]. However, FPGAs have certain limitations, including constrained on-chip resources. Moreover, adapting to new usage scenarios typically requires a hardware redesign.

Previous studies have sought to improve affine transformation efficiency through both algorithmic optimization and hardware acceleration techniques [15,16]. In the inseparable implementation method, the coordinate rotation digital computer (CORDIC) algorithm was used to design dedicated chips for implementing image rotation applications [17,18]. Although this algorithm does not require multiplication operations, the hardware requirements are higher, and the initial delay of the CORDIC is also higher. In the study by Bhandakar and Yu, an image rotation implementation method based on lookup tables (LUT) was reported, in which two independent LUTs were used to store the sine and cosine values for calculating the pixel positions in the image [19]. Owing to the movement of the center position of the image, each pixel position has a corresponding position in the image. In the study by Biswal and Banerjee, the proposed ATPR algorithm leverages the repetitive nature of pixel positions to perform parallel computations for the transformation of two pixel positions [20]. Biswal et al. proposed a modified ATPR (MATPR) algorithm that can calculate the transformation of four pixel positions through a single transformation operation [21]. Affine transformation by removing multiplications (ATRM) eliminates multiplications by leveraging the relationships between neighboring pixels but incurs significant overheads due to slow image data access speeds [22,23]. However, ATRM suffers from slow data access, and block methods often incur high bus loads because of the noncontiguous output addresses.

Hardware accelerators dedicated to affine transformations are typically implemented exclusively on FPGA. The exceptional parallel processing capability inherent to FPGA facilitates the concurrent computation of multiple pixel points throughout the affine transformation process. However, for complex systems, relying solely on FPGAs is insufficient to meet all task requirements. SoC architectures are more adept at performing system control-oriented tasks [24,25]. However, during the execution of an affine transformation, the SoC requires access to double data rate (DDR) memory via an advanced extensible interface (AXI), which leads to a decrease in the efficiency of the affine transformation operation [26]. Belokurov used high-level synthesis (HLS) to deploy affine transformations, with the process also utilizing the AXI interface and DMA [27]. Although the block data are successfully read, the writing process is still carried out address by address, which leads to a decrease in efficiency.

**Figure 1:**
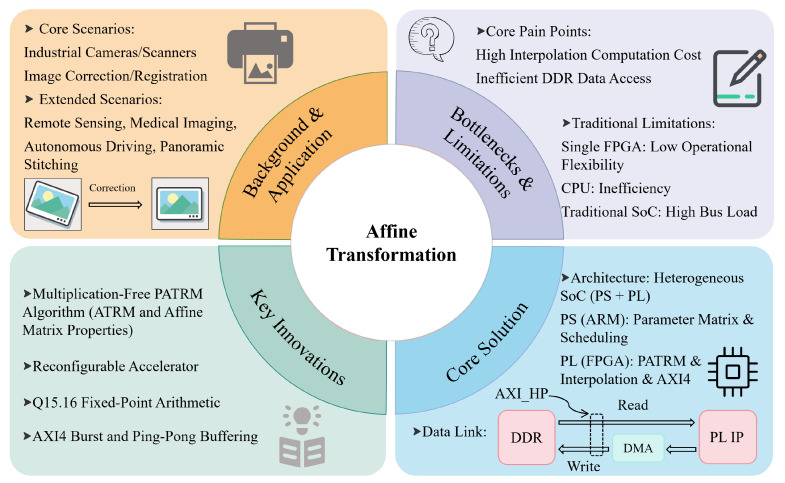
Research framework of the heterogeneous SoC-based FPGA accelerator for real-time affine transformation. The framework illustrates the logical relationship between application demands, technical bottlenecks, core solutions and key innovations, highlighting the problem-oriented design of the proposed accelerator.

Figure 1 illustrates the proposed solution to the bottlenecks of traditional affine transformation methods. We propose a heterogeneous SoC-based accelerator for affine transformation using FPGA technology. The architecture decouples the computation into control and data paths. The ARM in the PS handles control tasks (scheduling and parameter matrix generation), whereas the FPGA in the PL implements the proposed prefetch ATRM (PATRM) algorithm. PATRM integrates the multiplication-free method of ATRM with affine matrix properties, and AXI4 burst transmission optimizes DDR access for efficient prefetching and pipelined processing. The experimental results demonstrate that the proposed architecture achieves twice the throughput of the conventional solutions. The system meets the real-time requirement of 25 FPS for 2095×2448 image processing while providing computational determinism and maintaining system reconfigurability.

## 2. Algorithm Design

### 2.1. Proposed PATRM Algorithm

Affine transformations encompass fundamental geometric operations, such as rotation, scaling, translation, and shearing. The generalized 2D affine transformation is defined as [28]:

(1) $$\left[\begin{array}{c} x\\ y\\ 1 \end{array}\right]=\left[\begin{array}{ccc} b_{11} & b_{12} & b_{13}\\ b_{21} & b_{22} & b_{23}\\ 0 & 0 & 1 \end{array}\right]\left[\begin{array}{c} u\\ v\\ 1 \end{array}\right].$$

In Equation (1), (x,y) denotes the coordinates of a pixel in the source (input) image, and (u,v) denotes the coordinates of the corresponding pixel in the transformed (output) image. The coefficients $b_{11}$, $b_{12}$, $b_{13}$, $b_{21}$, $b_{22}$ and $b_{23}$ are the parameters that define a specific affine transformation.

During the inverse transformation process, the pixel positions in the input image are calculated on a pixel-by-pixel basis according to the spatial coordinates of the transformed image. The pixel values were obtained using the selected interpolation method and stored in the corresponding positions of transformed image. Equation (1) can be decomposed into two sub-equations, as shown in Equations (2) and (3):

(2) $$\begin{array}{c} x=b_{11}u+b_{12}v+b_{13}, \end{array}$$

(3) $$\begin{array}{c} y=b_{21}u+b_{22}v+b_{23}. \end{array}$$

In the ATRM, the coordinates (x,y) are derived from (u,v) using Equations (2) and (3). The subsequent pixel (u,v+1) is represented as $\left(x+b_{12},y+b_{22}\right)$. This recurrence enables the computation of multiple pixel coordinates with only one multiplication operation. With the ATRM used to reduce multiplications, this study further analyzes Equation (2). The pixel reading process is generally performed in a row-by-row manner, where the current row of pixels is processed before the next row. By setting the left-hand side of Equation (2) to x+1, we denote the row variation of the input image, thereby deriving the following equation:

(4) $$x+1=b_{11}u+b_{12}v_{0}+b_{13}.$$

Equation (4) is derived by considering a unit increment in the source row coordinate *x*, while keeping the transformation matrix coefficients unchanged. It reflects the mapping relationship between consecutive rows in the source and transformed images under the same affine matrix. The transition from (u,v) to $\left(u,v_{0}\right)$ involves a series of points that require interpolation, and the number of these points is determined by Δv. Subtracting Equation (3) from Equation (4) yields Δv:

(5) $$\Delta v=v_{0}-v=\frac{1}{b_{12}}.$$

Note that Equation (5) holds under the assumption that $b_{12}\neq 0$, which is generally true for transformations involving rotation or shearing. For pure scaling along axes ($b_{12}=0$), the mapping degenerates to a row-wise independent case, and the burst length is determined by the image width. Unlike ATRM, which suffers from scattered memory access, PATRM introduces prefetching by leveraging the row-wise spatial correlation in the source image. This allows continuous burst reads from DDR, reducing access overhead while retaining the multiplication-free recurrence.

**Figure 2:**
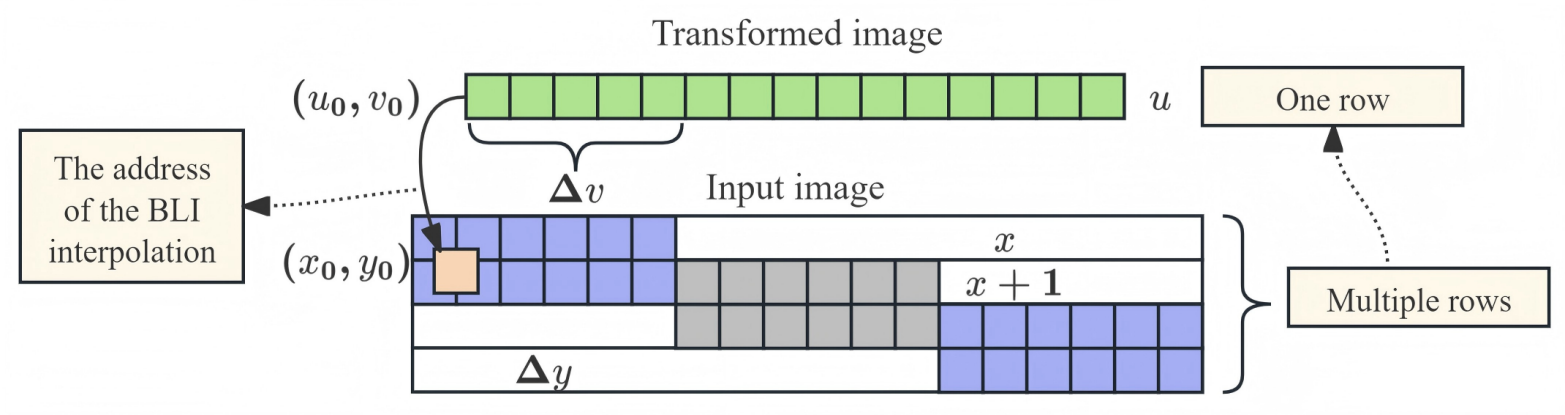
Principle of row-wise affine transformation with BLI. Multiple rows from the source image are prefetched based on the calculated span Δv to generate one row of the transformed image. This illustrates the spatial locality exploited by PATRM for efficient AXI4 burst reading.

For a range of Δv in the transformed image, the corresponding pixels in the input image are in the same row. The pixels within the same row of the transformed image (*u* unchanged) are mapped to spatially correlated pixels in the source image that share the same row indices. This spatial correlation implies that the source pixel data required for processing a transformed row are located in contiguous memory rows. For a unit increment in the transformed row coordinate *u*, the corresponding change in the source column coordinate *x*, denoted by Δy, can be computed. As shown in Figure 2, within a span Δv, the mapped source image pixels occupy identical row coordinates. The value Δy determines the optimal burst length for the AXI4 burst transmission mechanism, thereby facilitating efficient and contiguous data access for the ATRM interpolation process.

### 2.2. BLI Algorithm

**Figure 3:**
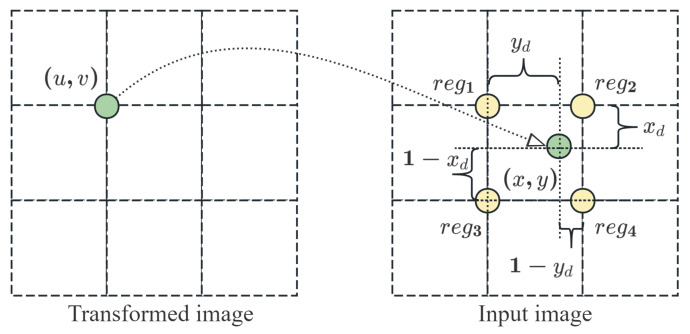
Process of address mapping. Mapping results of the address determines the interpolation parameters.

In the address mapping process, the input pixel addresses (x,y) are often non-integer values. Interpolation techniques must be employed to ensure that non-integer addresses obtain accurate pixel values. The commonly used interpolation methods are nearest-neighbor interpolation (NNI) and bi-linear interpolation (BLI). To ensure the image quality of the affine transformation, we employed BLI to implement the proposed algorithm. Figure 3 illustrates the address mapping process and its relationship with the four surrounding grid points required for the BLI.

To calculate the BLI, it is necessary to obtain the values of the four pixels surrounding the non-integer points in the image. (u,v) represents the transformed pixels mapped to a non-integer point in the input image. To calculate the pixel value of point (u,v) using BLI, it is necessary to obtain the pixel values of the four grid points $reg_{1}$, $reg_{2}$, $reg_{3}$ and $reg_{4}$. Using equation:

(6) $$\begin{array}{cc} D= & x_{f}y_{f}reg_{4}+\left(1-x_{f}\right)y_{f}reg_{2}+x_{f}\left(1-y_{f}\right)reg_{3}+\left(1-x_{f}\right)\left(1-y_{f}\right)reg_{1}, \end{array}$$

the pixel value D of (u,v) can be calculated. The $\left(x_{f},y_{f}\right)$ in the equation represents the fractional part of (x,y).

### 2.3. Fixed-Point Operations

Traditional affine transformation algorithms use floating-point operations. Parameters are floating-point numbers, whereas pixel data are integers, thus requiring post-processing to convert the transformed floating-point pixel data into integers. Their floating-point computations result in high resource consumption and low throughputs. To reduce complexity and improve efficiency, the accelerator proposed in this study uses fixed-point arithmetic. In FPGAs, fixed-bit-width data are directly generated via variable declarations and transformed pixels through bit truncation. With the 32-bit AXI_Lite interface, the transformation parameters use a Q15.16 (INT16) format, plus one sign bit.

## 3. Hardware Design

### 3.1. System Architecture

**Figure 4:**
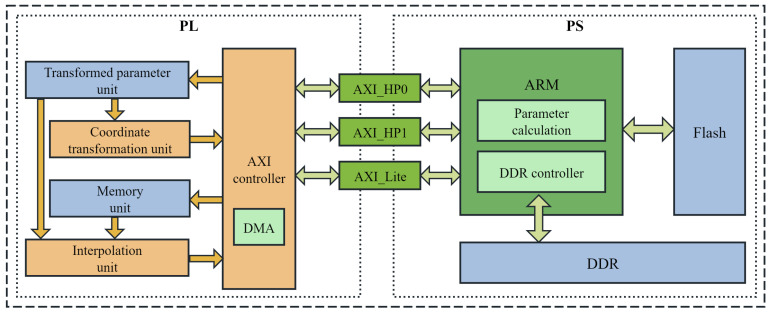
System architecture.

A block diagram of the accelerator system is shown in Figure 4. The pipelined computation of the affine transformation, which is the core of the entire hardware accelerator, handles the pixel prefetching and interpolation. Efficient block data prefetching is achieved through AXI4 burst transmission, where contiguous pixel rows in the source image are read in advance based on the calculated Δv range. In data transmission, PATRM executes only one read and one write operation per data block. In contrast, Block AT typically performs one read followed by multiple writes. This single read-write scheme reduces the total number of DDR accesses, which aligns with the spatial locality exploited by PATRM.

The PL section incorporates four core computational Intellectual Property (IP) blocks, including the transformed parameter unit, coordinate transformation unit, memory unit, and interpolation unit. These units are pipelined based on the pixel data flow. The AXI controller manages the initialization of the parameters and distribution of tasks to the computational units. A Direct Memory Access (DMA) controller handles high-speed data transfer between the PL (FPGA) and PS (ARM core). Specifically, the DMA controller reads the source images from the DDR and writes the transformed images to the DDR. The PS primarily consists of an ARM processor, DDR memory, and a flash storage. The affine transformation matrices are stored in flash memory, and the ARM processor orchestrates the entire transformation process.

During global data scheduling within the system, data communication between the PS and PL is achieved through two AXI_HP interfaces and one AXI_Lite interface. In our implementation, the transformation matrix and image information are first written to the transformation parameter unit using the AXI_Lite interface. Once the configuration was complete, a start command was given. The coordinate transformation unit then calculates and reads the address information. Subsequently, the pixels were stored in the RAM-based memory unit through the AXI_HP interface. Finally, the interpolation unit completed the interpolation. The DMA transfers the processed pixels back to the DDR through AXI_HP interface.

### 3.2. Hardware Module Design

The design of the PL portion of the system was divided into two main components. The first component comprises the control and configuration paths. It is primarily responsible for system scheduling and parameter configuration of various modules, such as the affine matrices and image data. The second component is the data path. It encompasses the computational process of affine transformation from an input image to an output image.

During the affine transformation process, the transformed parameter unit uses several registers to cache the configuration parameters from the PS and forward the commands. The key parameters include the image storage address, image information, and the start and stop commands. Once the configuration information is ready, a start command is issued. The transformation parameter unit loads various parameters into the downstream units to initiate an affine transformation process. Affine transformations of images of different sizes can be performed using a dynamic parameter configuration.

**Figure 5:**
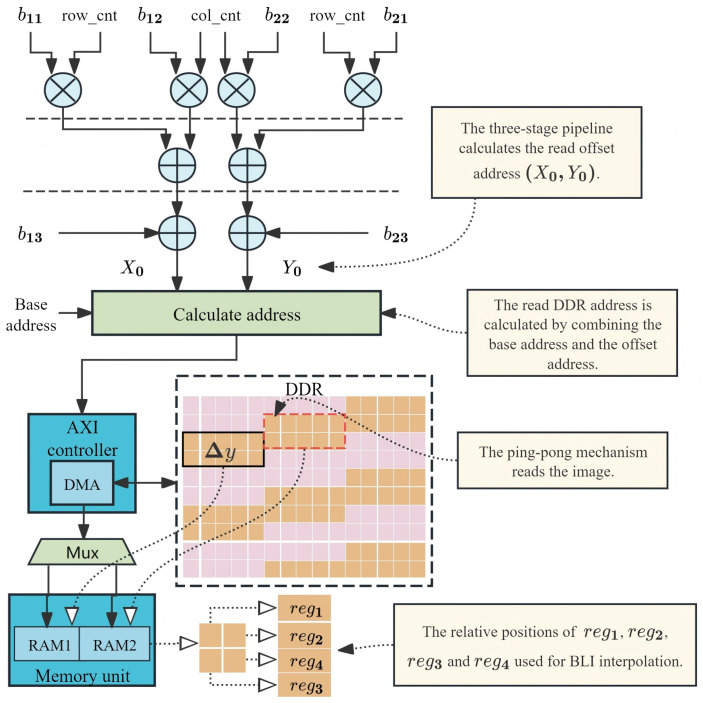
Pipeline for pixel address calculation and DDR read control. The coordinate transformation unit generates addresses in a three-stage pipeline. The burst_len signal, derived from Δy, controls the AXI4 master to perform burst reads. A ping-pong buffer (RAM1/RAM2) allows concurrent data prefetching and interpolation.

After the start command is received, the coordinate transformation unit converts the position of the target pixel to the corresponding position of the source pixel. It cooperates with the AXI controller to complete the reading of pixel data, as shown in Figure 5. The initial coordinates of the source pixel were calculated using the proposed PATRM algorithm. The system then uses a burst transmission mechanism to read the gray values of the pixels in the same source row into memory units. Based on the row and column positions of the current output image, an affine transformation matrix was used to calculate the position of the input image. A three-stage pipeline was adopted to complete these calculations and accelerate the operating frequency. The read address of the DDR is jointly determined by the base address and coordinates.

With traditional DDR reading methods directly deployed on an FPGA using serial computing hardware platforms, different addresses in the DDR are accessed frequently. This not only wastes hardware resources but also limits the execution speed of the hardware. The proposed PATRM determines the burst-transmission length. It can simultaneously read multiple pixels. Data read from the DDR are stored in the RAM through a selector for interpolation calculations. In the interpolation process, the data were written to RAM1 for interpolation calculations. Meanwhile, PATRM predicts the next burst transmission address using Δy and $\left(X_{0},Y_{0}\right)$. The read data were then written to RAM2. Subsequent operations were performed using a ping-pong mechanism.

**Figure 6:**
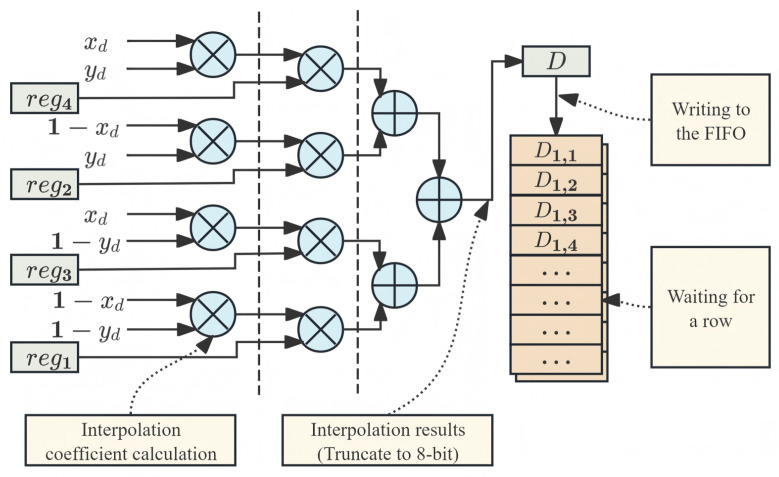
Four-stage pipelined interpolation and output caching. Stage 1 calculates coefficients. Stage 2 coefficient is multiplied by the pixels. Stage 3 and 4 sum the results. The AXI_HP interface writes the FIFO of DMA contents back to DDR in bursts.

When the data are stored in the memory unit, the coordinate transformation unit performs interpolation. The interpolation calculation process is illustrated in Figure 6. The process of calculating the pixel values also adopts a four-stage pipeline. The interpolation weights for the four pixels were determined using the fractional component $\left(X_{0},Y_{0}\right)$ calculated by the coordinate transformation unit. The data read from the RAM underwent interpolation. To reduce the computational complexity, the pipeline truncates the data-bit width. Because the pixel data are integers, truncating the fractional part introduces a negligible error while significantly increasing the operating speed. The interpolated result *D* is initially buffered in the FIFO. Upon reaching the maximum burst length, the FIFO contents were written to the specified location in the DDR using the AXI_HP interface.

## 4. Experimental

**Figure 7:**
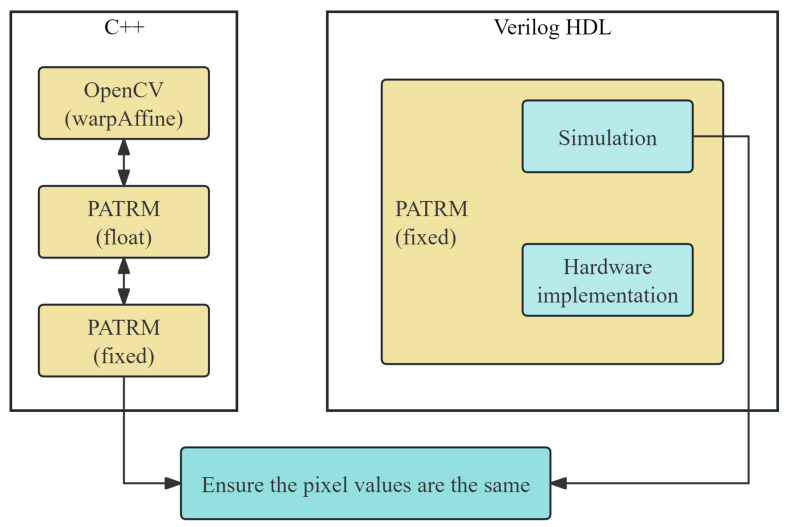
Algorithm verification process. Gradually verify to ensure the correctness of the algorithm.

The complete algorithm verification process is illustrated in Figure 7. The feasibility of the algorithm was ensured by using C++ 17 to implement OpenCV, floating, and fixed PATRM for cross-validation. The results showed that the proposed PATRM can perform image affine transformations. Subsequently, the quantified PATRM was implemented using Verilog HDL and deployed on the designated platform for simulation and hardware deployment. This algorithm verification process, which started with software and progresses through simulation to hardware, ensured the accuracy of the algorithm results. The test images included a 256×256 photographer image and a series of images, all represented by 256 grayscale levels.

### 4.1. Simulation Results

**Figure 8:**
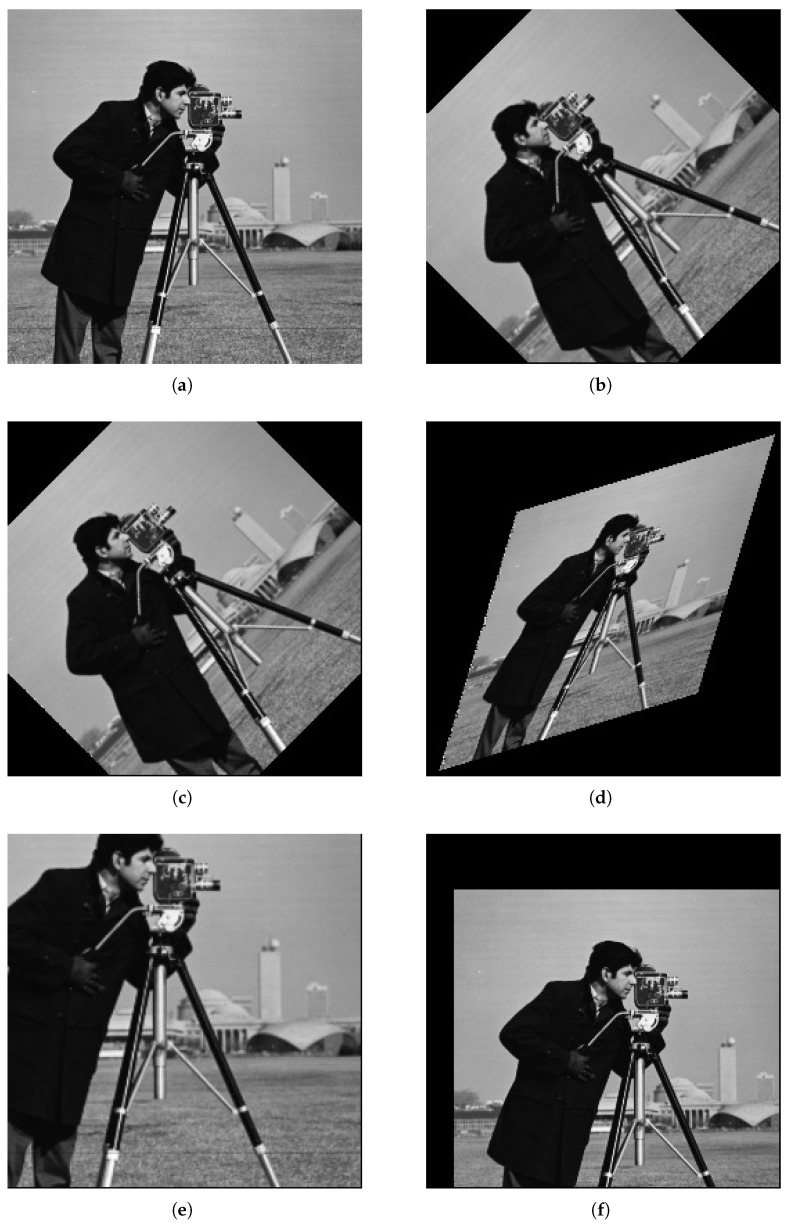
The proposed PATRM algorithm in C++ for different affine transformations: (**a**) original image, (**b**) rotation by 45° using a conventional algorithm, (**c**) rotation by 45° using the PATRM algorithm, (**d**) shearing, (**e**) scaling, (**f**) translation.

Figure 8a shows the original image, whereas Figure 8b,c illustrate the affine rotation operations performed using the OpenCV method and the proposed PATRM algorithm, respectively. Figure 8d–f demonstrate the transformation effects of shearing, scaling, and scaling plus translation. The sample image was a 256×256 photographer image. These results indicate that the visual image quality remains unchanged by the proposed PATRM algorithm.

Table 1 quantifies the execution times of the three algorithms. The algorithms used were OpenCV, floating-PATRM, and fixed-PATRM. The evaluation covered different image sizes and hardware platforms. This table enables a quantitative evaluation of the time efficiency of the algorithms at the software level. For a small size 256×256 image on the CPU, the fixed-PATRM algorithm achieved an execution time of 2.09 ms. It is 9.5% faster than the floating-PATRM algorithm, with an execution time of 2.31 ms. The speedup is attributed to the reduced computational complexity of fixed-point arithmetic. The fixed-PATRM algorithm is only 10.6% slower than the optimized OpenCV implementation, which has an execution time of 1.89 ms. This gap is negligible. The algorithm features a hardware-oriented design. As the image resolution scales to large formats, two typical examples are 2095×2448 and 5695×7344. The advantage of fixed-PATRM becomes more pronounced when using a CPU. For the two large sizes, fixed-PATRM outperforms floating-PATRM by 9.4% and 9.3%, respectively. The time cost of fixed-PATRM remains close to that of OpenCV.

**Figure 9:**
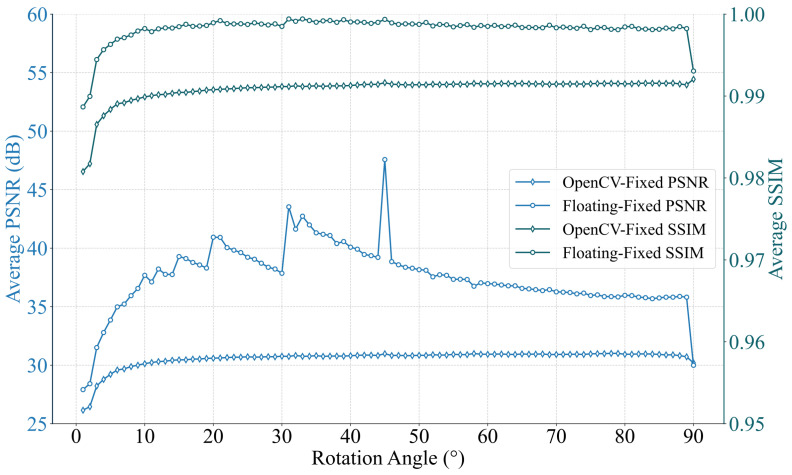
Image quality comparison of the fixed-point PATRM algorithm. The PSNR and SSIM are calculated by using the results from OpenCV warpAffine function and the ARM-based floating-point PATRM software implementation as the reference, respectively. The term “OpenCV-Fixed” denotes the comparison between fixed-point PATRM and OpenCV reference; “Floating-Fixed” denotes the comparison between fixed-point and floating-point PATRM.

When deployed on the PS of the SoC (ARM core), the fixed-PATRM consistently demonstrated a lower latency than the floating-PATRM. For 2095×2448 images, the latency values were 157.68 ms for fixed-PATRM and 173.89 ms for floating-PATRM. These results verified the Q15.16 fixed-point optimization. It effectively mitigates the computational overhead of the affine transformation on embedded processing cores. This higher latency can be attributed to the lower clock frequency of the ARM core and DDR memory access constraints via the AXI interface. Subsequent FPGA-based acceleration in the PL section aims to overcome these limitations. The fixed-PATRM algorithm preserves the image quality, as shown in Figure 9. In addition, it achieves superior computational efficiency compared with its floating-point counterpart at the software level. This algorithm lays a solid foundation for high-performance hardware implementation on heterogeneous SoC.

### 4.2. Image Quality Analysis

The image quality of the fixed-point PATRM implementation was evaluated against two benchmarks: the OpenCV affine transformation and floating-point implementation of the PATRM algorithm. The PSNR was computed for rotations ranging from 0° to 90° in 1° increments. PSNR was calculated using equation [29]:

(7) $$\begin{array}{c} PSNR=10\log_{10}\left(\frac{{MAX}^{2}}{MSE}\right). \end{array}$$

In Equation (7), MAX represents the maximum possible pixel value (e.g., 255 for 8-bit grayscale images). The Mean Squared Error (MSE) between the reference image ($I_{1}$) and processed image ($I_{2}$) is given by the following equation:

(8) $$\begin{array}{c} MSE=\frac{1}{MN}\sum_{i=1}^{M}\sum_{j=1}^{N}{\left[I_{1}\left(i,j\right)-I_{2}\left(i,j\right)\right]}^{2}, \end{array}$$

where M and N denote the height and width of the image in pixels. $I_{1}\left(i,j\right)$ and $I_{2}\left(i,j\right)$ represent the pixel intensity values at locations (i,j) in $I_{1}$ and $I_{2}$, respectively.

To evaluate the image quality of our fixed-point hardware implementation, we computed the PSNR and Structural Similarity (SSIM) indices using the OpenCV warpAffine function (double-precision floating-point) and our own floating-point PATRM algorithm implementation software references as the ground truth. The results, labeled as ’OpenCV-Fixed’ and ’Floating-Fixed’ respectively, are plotted against rotation angles in Figure 9.

Figure 9 shows that the PSNR value of the proposed algorithm matches well with those of the OpenCV and floating-point PATRM algorithms for BLI. The PSNR value of the proposed algorithm ranged between 26 and 30 dB, remaining stable at approximately 30 dB for angles exceeding 10°. The fixed-point PATRM maintains an average SSIM above 0.980 when compared to the OpenCV benchmark, and above 0.988 compared to the floating-point PATRM implementation across all rotation angles. Even at small angles (0°–10°) where quantization effects are more pronounced, the SSIM remains above 0.98, indicating well-preserved structural fidelity.

At small angles, the PSNR slightly deteriorated. Because the Structural Similarity (SSIM) decreases at small angles, the main reason for the reduction in PSNR is the error in the image structure, rather than a real decline in image quality. Further analysis shows that when the angle approaches 0°, sinθ≃θ becomes very small. For example, for 1°, the Q15.16 fixed-point representation is 0.017453×65536=1143. The relative proportion of the quantization error (±1) was 1÷1143=0.087% (5° is 0.017%), which was significantly larger than that of the error at medium and high angles. This amplifies the deviations in the coordinate calculations and increases the errors in the interpolated pixels, which are relatively high. During the interpolation process from point (u,v) to point (u,v+1), the fixed-point PATRM introduces quantization errors that differ from the rounding errors inherent in floating-point PATRM. Nevertheless, when compared to the results generated by OpenCV warpAffine function, the images processed by fixed-point PATRM maintain a PSNR above 26 dB, confirming that the introduced errors remain within an acceptable range for industrial imaging applications.

In the context of industrial imaging for tasks such as scanner correction and alignment, a PSNR above 26 dB is widely regarded as acceptable for maintaining functional accuracy [21,30,31]. The proposed fixed-point PATRM consistently exceeds this practical threshold (PSNR > 26 dB, SSIM > 0.98) across all transformations, confirming its suitability for real-time industrial applications where a balance between computational efficiency and image quality is paramount [32,33].

### 4.3. Implementation Results

The hardware platform used in this experiment was an Anlogic DR1M90GEG400 development board. The PL section of the board was equipped with a high-performance FPGA. In this study, an affine transformation accelerator was designed. The operating frequency of the accelerator was 200 MHz. The proposed architecture was implemented directly on the FPGA of the SoC system using Verilog HDL.

Table 2 presents the FPGA resource utilization of the proposed PATRM accelerator. The accelerator was synthesized and implemented on an Anlogic DR1 development board. The results demonstrate the high resource efficiency of the proposed architecture. Specifically, the design consumes only 2934 look-up tables (LUT), which accounts for 5.59% of the 52,480 available resources. This low utilization originates from two key optimizations. The multiplication-free design of the PATRM algorithm and the adoption of pipelined logic, both of which enhance the efficiency of the coordinate transformation and interpolation circuits. Regarding the embedded random access memory (ERAM), 66 units were utilized, representing 23.57% of the 280 available units. These units mainly serve to cache pre-fetched pixel blocks and the intermediate interpolation results. The AXI4 burst transmission mechanism reduces redundant storage requirements. The usage of digital signal processors (DSP) is minimal at 17 units, which constitutes 7.08% of the 240 available DSPs. Multiplications in Equations (2) and (3) are eliminated via recurrence relations between neighboring pixels. The 17 DSPs are used primarily for address calculation during the first read operation and interpolation calculations (Equation (6)). This low DSP consumption is enabled by Q15.16 fixed-point arithmetic. Complex floating-point operations are replaced with streamlined integer computations through this arithmetic. This replacement reduces the demand for dedicated DSP resources.

**Figure 10:**
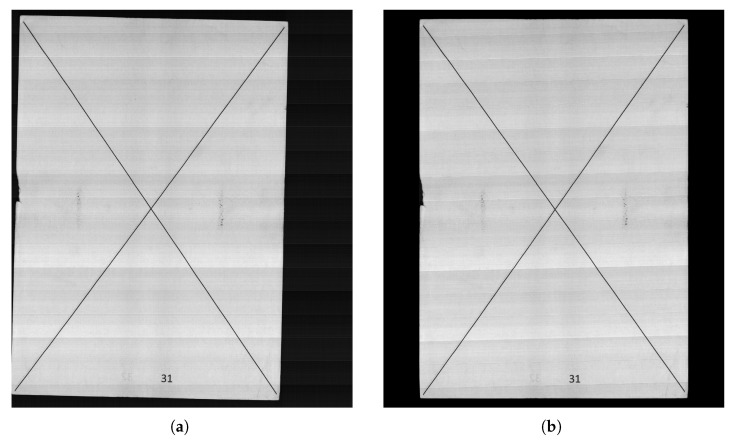
The results of the proposed PATRM scanning and correction algorithm in 2095×2448 format image: (**a**) the scanned original image, (**b**) the image after PATRM correction.

The proposed PATRM was deployed in an industrial scanner. During the operation of the scanner, slight movements or vibrations of the machine can cause the position of the scanned image to be shifted. To obtain an image in the correct position, centering and rotation operations are required. The PS controls the scanning process and calculation of the transformation matrix and sends the correction commands to the PL through the AXI interface. This achieves image correction at 25 FPS in 2095×2448 format. The scanning and correction results are presented in Figure 10. The process of converting the original image into a correctly positioned image was completed.

Table 3 compares the performance of the proposed PATRM algorithm with that of traditional affine transformation algorithms. Two key dimensions are involved in this process. They are the processing rate, expressed as pixels per second (pixel/s), and hardware architecture. Most traditional algorithms adopt a single FPGA or CPU architecture, whereas only the Block AT algorithm and the proposed PATRM rely on the SoC architecture. Most traditional single-architecture algorithms suffer from low processing efficiencies. For example, the CORDIC algorithm proposed by Jiang et al. runs on the XC2V1000 FPGA, achieving only 4.45 M pixel/s. The CATM algorithm proposed by Lee et al. operates on a CPU, with a performance of 18.72 M pixel/s. The solution proposed by Hernandez et al. was implemented on a Spartan3E1600 FPGA, achieving 39.32 M pixel/s. All these single-architecture methods often generate non-continuous output image addresses, which increase the bus load and restrict system speed. The Block AT algorithm, while implemented on a modern SoC platform, exhibits relatively high LUT (11,000) and BRAM (1800 Kb) consumption for its achieved performance of 24.32 M pixel/s. However, it did not list the usage amount of DSP. In contrast, the proposed PATRM runs on a DR1M90GEG400 based SoC architecture. It achieves a processing rate of 128.21 M pixel/s, far exceeding both traditional single-architecture algorithms and the Block AT (SoC) algorithm. This superior performance stems from the targeted optimization of the PATRM for the SoC characteristics. The linear algebraic properties of transformation matrices are leveraged by PATRM. Continuous data rows are output by the PATRM. This optimizes data transmission under the AXI interface of the SoC. The computation efficiency under the DDR access mechanism is also improved.

Among all the compared methods, only the MATPR algorithm by Biswal et al. achieves a higher throughput (1129.93 M pixel/s). The implementation of multipliers using slices, as opposed to dedicated DSP blocks, leads to higher slice utilization. This peak performance is attained through a deeply pipelined, parallel architecture that employs direct and fixed-pattern DDR data reading. However, this approach increases hardware resource requirements and demands a memory access pattern that is often optimized for specific image dimensions and transformation parameters. Consequently, adapting to images of different sizes or affine transformations typically necessitates hardware redeployment (re-synthesis), resulting in low operational flexibility.

In contrast, the proposed PATRM, architected for the heterogeneous SoC platform, prioritizes a balance between performance, flexibility, and resource efficiency. The high RAM usage in PATRM is attributed to the preallocated space for accommodating images of varying sizes and the buffer occupancy required for DMA operations. While its throughput (128.21 M pixel/s) is lower than MATPR peak, it sustains real-time performance for target applications. Crucially, PATRM design leverages the spatial locality of affine transformations to prefetch contiguous data via parameterized AXI4 burst transmission. This allows the PS to dynamically calculate and output different affine matrices according to application needs. The PATRM accelerator in the PL can then adapt to these new parameters in real-time without hardware redesign or reconfiguration. This inherent flexibility enables seamless support for diverse affine operations and variable image sizes, outperforming most traditional single FPGA/CPU solutions and the existing SoC-based Block AT algorithm. Thus, this study demonstrates that PATRM provides an effective, superior, and highly adaptable solution for practical industrial imaging scenarios where both performance and adaptability are required.

### 4.4. Latency and Scalability Analysis

To provide a comprehensive understanding of the operational characteristics of this system, an analysis of latency composition and architectural scalability is presented. The overall latency for processing a 2095×2448 image arises from both the PS and the PL. The PS contributes approximately 10% of the total latency, which primarily includes the time for the ARM core to compute the affine transformation matrix and, more significantly, to configure the PL parameters and initiate DMA transfers via the AXI_Lite interface. This control overhead remains relatively constant across different image sizes after initial setup. The dominant portion (90%) stems from the PL, encompassing the complete PATRM computation pipeline: coordinate address generation, burst data prefetching from DDR via AXI_HP, the bilinear interpolation calculation, and the final write-back of results. This distribution validates the efficacy of the heterogeneous architecture, which successfully offloads the computationally intensive, deterministic pixel processing to the parallel PL while retaining flexible control in the PS.

The proposed accelerator architecture is inherently scalable to accommodate more complex imaging requirements. For multi-channel color images such as RGB, the most straightforward extension involves replicating the grayscale processing datapath, comprising the coordinate transformation, prefetch logic and interpolation unit for each color channel. This channel-parallel approach would leverage the same shared control logic and AXI/DMA infrastructure, primarily requiring an adjustment to the data path width and a proportional increase in DDR bandwidth, for which the AXI4 burst mechanism remains equally efficient. To handle image data with higher bit depths such as 10, 12 or 16 bits, the Q15.16 fixed-point arithmetic core can be adapted by extending the integer part of the format (for example, extending to Q17.16) while preserving the fractional precision for interpolation. Such an adjustment would have a minimal impact on LUT utilization but might necessitate wider internal datapaths and could moderately increase DSP usage for the interpolation multiplications. These clear pathways for extension underscore the potential of this design potential to meet the demands of advanced industrial applications involving color or high dynamic range content without necessitating a fundamental architectural redesign.

## 5. Conclusions

This study presents a PATRM accelerator architecture based on a heterogeneous SoC. By decoupling the control and data paths, employing a multiplication-free design, and utilizing AXI4 burst prefetching, the system achieves real-time affine transformation at 25 FPS for images with a resolution of 2095×2448, striking a favorable balance between throughput and resource efficiency. The system supports real-time affine transformation of images with varying dimensions through dynamic parameter configuration. This capability for real-time processing of images of variable sizes highlights the versatility and computational efficiency of the architecture. This study provides an efficient and reconfigurable hardware solution capable of fulfilling the real-time requirements of affine transformation in image processing applications [34,35,36].

However, this work has several limitations, which point to clear directions for future research. First, the adopted Q15.16 fixed-point arithmetic leads to a noticeable drop in PSNR at very small rotation angles due to the increased relative proportion of quantization error. Second, the performance of the system is heavily relies on contiguous burst data transmission via the AXI4 interface. In practice, if the source image data lacks ideal storage continuity, the efficiency of the prefetching mechanism would be compromised. Furthermore, while the current architecture is primarily validated for grayscale images and possesses a theoretically scalable path for color image processing, its actual throughput and resource consumption under multi-channel data flow require further evaluation.

To address these limitations and further enhance the system, future work will focus on optimizing the AXI4 burst transmission mechanism and on-chip buffer capacity to improve adaptability to diverse data layouts and achieve even faster real-time performance. Advanced interpolation algorithms such as bicubic interpolation will be integrated into the PATRM framework, coupled with targeted hardware optimizations to better balance image quality and computational overhead. Finally, a portable IP core compatible with mainstream SoC/FPGA platforms will be developed to extend the practicality and scalability of this accelerator in broader industrial and consumer electronics applications.

## Figures and Tables

**Table 1:** The total time (ms) spent by different affine transformation algorithms using C++.

Image Size	Device	Algorithm		
		OpenCV	Floating-PATRM	Fixed-PATRM
256×256	CPU	1.89	2.31	2.09
2095×2448	CPU	79.77	97.07	87.92
	SoC(PS)	N/A	173.89	157.68
5695×7344	CPU	596.21	721.29	654.04
	SoC(PS)	N/A	1402.94	1272.14

**Table 2:** Synthesis and implementation results.

Resource	Used	Available	Utilization (%)
LUT	2934	52,480	5.59%
ERAM	66	280	23.57%
DSP	17	240	7.08%

**Table 3:** Comparison of the Affine operation.

Reference	Algorithm	Device	LUT	DSP	Slice	RAM (Kb)	Performance (pixel/s)
Jiang et al. [18]	CORDIC	XC2V1000	663	2	420	576	4.45 M
Lee et al. [23]	CATM	CPU	-	-	-	-	18.72 M
Hernandez et al. [6]	-	Spartan3E1600	-	8	995	54	39.32 M
Biswal et al. [21]	MATPR	XC2VP30-6FF676	2283	-	1622	576	1129.93 M
Belokurov [27]	Block AT	XC7VX485TFFG	11,000	-	-	1800	24.32 M
Proposed	PATRM	DR1M90GEG400	2934	17	891	1320	128.21 M

## Data Availability

The data presented in this study are available on request from the corresponding author.

## References

[1] Modenov P., Parkhomenko A. (1965). Affine Transformations. Euclidean and Affine Transformations.

[2] Gao J., Sun Z. (2022). An Improved ASIFT Image Feature Matching Algorithm Based on POS Information. Sensors.

[3] Lian T., Pei D., Qiang L., Alsmadi M. (2013). Fast Correction Algorithm Research of Image Geometric Distortion in the Image Tracking. J. Appl. Sci..

[4] Manmatha R. Image matching under affine deformations. Proceedings of the 27th Asilomar Conference on Signals, Systems and Computers.

[5] Gee J.C., Reivich M., Bajcsy R. (1993). Elastically Deforming 3D Atlas to Match Anatomical Brain Images. J. Comput. Assist. Tomogr..

[6] Jereb Z., Diaci J. (2010). Real-time geometrical correction of video image using FPGA. IEICE Electron. Express.

[7] Zitová B., Flusser J. (2003). Image registration methods: A survey. Image Vis. Comput..

[8] Melo R., Barreto J.P., Falcao G. (2012). A New Solution for Camera Calibration and Real-Time Image Distortion Correction in Medical Endoscopy–Initial Technical Evaluation. IEEE Trans. Biomed. Eng..

[9] Lin H., Du P., Zhao W., Zhang L., Sun H. Image registration based on corner detection and affine transformation. Proceedings of the 2010 3rd International Congress on Image and Signal Processing.

[10] Shams R., Sadeghi P., Kennedy R., Hartley R. (2010). A Survey of Medical Image Registration on Multicore and the GPU. IEEE Signal Process. Mag..

[11] Zecchino V., Lombardi L., Marzocca C., Patimisco P., Sampaolo A., Spagnolo V.L. (2025). Development of Compact Electronics for QEPAS Sensors. Sensors.

[12] Peng X., Fan H., Zhou W. Digital Image Processing System Based on FPGA. Proceedings of the 2023 5th International Conference on Circuits and Systems (ICCS).

[13] Yang Z. (2025). Saturation Adjustment of Image Enhancement Based on FPGA. Appl. Comput. Eng..

[14] Lakshmi C., Sri Y.D., Subashini R., Vinizia, Amirtharajan R., Thanikaiselvan V., Mahalingam H. Implementation of Chaos-Based Image Encryption on FPGA. Proceedings of the 2023 2nd International Conference on Vision Towards Emerging Trends in Communication and Networking Technologies (ViTECoN).

[15] Hernandez A., Gardel A., Perez L., Bravo I., Mateos R., Sanchez E. Real-Time Image Distortion Correction using FPGA-based System. Proceedings of the IECON 2006—32nd Annual Conference on IEEE Industrial Electronics.

[16] Pflugfelder D., Scharr H. (2020). Practically Lossless Affine Image Transformation. IEEE Trans. Image Process..

[17] Sahoo A., Panigrahy M. Hardware Implementation of CORDIC Algorithm. Proceedings of the 2018 International Conference on Applied Electromagnetics, Signal Processing and Communication (AESPC).

[18] Jiang X.G., Zhou J.Y., Shi J.H., Chen H.H. FPGA implementation of image rotation using modified compensated CORDIC. Proceedings of the 2005 6th International Conference on ASIC.

[19] Bhandakar S., Yu H. VLSI implementation of real-time image rotation. Proceedings of the 3rd IEEE International Conference on Image Processing.

[20] Biswal P., Banerjee S. An embedded solution of 2D fast affine transform for biomedical imaging systems. Proceedings of the VLSI Design and Test Symposium (VDAT 2009).

[21] Biswal P.K., Mondal P., Banerjee S. (2013). Parallel architecture for accelerating affine transform in high-speed imaging systems. J. Real-Time Image Process..

[22] Badawy W., Bayoumi M. (2002). A Multiplication-Free Algorithm and A Parallel Architecture for Affine Transformation. J. VLSI Signal Process. Syst. Signal Image Video Technol..

[23] Lee S., Lee G.G., Jang E.S., Kim W.Y., Hutchison D., Kanade T., Kittler J., Kleinberg J.M., Mattern F., Mitchell J.C., Naor M., Nierstrasz O., Pandu Rangan C., Steffen B. (2006). Fast Affine Transform for Real-Time Machine Vision Applications. Intelligent Computing.

[24] Yang D., Li J., Hao G., Chen Q., Wei X., Dai Z., Hou Z., Zhang L., Li X. (2024). Hardware accelerator for high accuracy sign language recognition with residual network based on FPGAs. IEICE Electron. Express.

[25] Kim H., Kim T.K. (2025). Design and Implementation of a YOLOv2 Accelerator on a Zynq-7000 FPGA. Sensors.

[26] Leung A. (2015). FPGA-based image edge detection IP core design. Engineering Management and Industrial Engineering.

[27] Belokurov V.A. Implementation of affine transform for image rotation using a HLS language. Proceedings of the 2018 7th Mediterranean Conference on Embedded Computing (MECO).

[28] Snapper E., Troyer R.J. (1971). Affine geometry. Metric Affine Geometry.

[29] Keles O., Yilmaz M.A., Tekalp A.M., Korkmaz C., Dogan Z. On the Computation of PSNR for a Set of Images or Video. Proceedings of the 2021 Picture Coding Symposium (PCS).

[30] Deshpande R.G., Ragha L.L., Sharma S.K. (2018). Video Quality Assessment through PSNR Estimation for Different Compression Standards. Indones. J. Electr. Eng. Comput. Sci..

[31] Dremin M., Kozhemyakov K., Molodetskikh I., Kirill M., Sagitov A., Vatolin D. (2024). Machine vision-aware quality metrics for compressed image and video assessment. International Conference on Pattern Recognition.

[32] Wang Z., Bovik A., Sheikh H., Simoncelli E. (2004). Image quality assessment: From error visibility to structural similarity. IEEE Trans. Image Process..

[33] Sara U., Akter M., Uddin M.S. (2019). Image Quality Assessment through FSIM, SSIM, MSE and PSNR—A Comparative Study. J. Comput. Commun..

[34] Fu J., Nie C., Sun F., Li G., Shi H., Wei X. (2024). Bionic visual-audio photodetectors with in-sensor perception and preprocessing. Sci. Adv..

[35] Jiang H., Fu J., Wei J., Li S., Nie C., Sun F., Wu Q.Y.S., Liu M., Dong Z., Wei X. (2024). Synergistic-potential engineering enables high-efficiency graphene photodetectors for near- to mid-infrared light. Nat. Commun..

[36] Fu J., Guo Z., Nie C., Sun F., Li G., Feng S., Wei X. (2024). Schottky infrared detectors with optically tunable barriers beyond the internal photoemission limit. Innovation.

